# Chronic Hypoxia Enhances β-Oxidation-Dependent Electron Transport via Electron Transferring Flavoproteins

**DOI:** 10.3390/cells8020172

**Published:** 2019-02-18

**Authors:** Dominik C. Fuhrmann, Catherine Olesch, Nina Kurrle, Frank Schnütgen, Sven Zukunft, Ingrid Fleming, Bernhard Brüne

**Affiliations:** 1 Institute of Biochemistry I, Faculty of Medicine, Goethe-University Frankfurt, 60590 Frankfurt, Germany; fuhrmann@biochem.uni-frankfurt.de (D.C.F.); olesch@biochem.uni-frankfurt.de (C.O.); 2 Department of Medicine, Hematology/Oncology, Goethe-University Frankfurt, 60590 Frankfurt, Germany; kurrle@med.uni-frankfurt.de (N.K.); schnuetgen@em.uni-frankfurt.de (F.S.); 3 German Cancer Consortium (DKTK), Partner Site, 60590 Frankfurt, Germany; 4 Frankfurt Cancer Institute, Goethe-University Frankfurt, 60596 Frankfurt, Germany; 5 Institute for Vascular Signaling, Faculty of Medicine, Goethe-University Frankfurt, 60590 Frankfurt, Germany; Zukunft@vrc.uni-frankfurt.de (S.Z.); fleming@em.uni-frankfurt.de (I.F.); 6 Project Group Translational Medicine and Pharmacology TMP, Fraunhofer Institute for Molecular Biology and Applied Ecology, 60596 Frankfurt, Germany

**Keywords:** mitochondria, electron transport chain, complex I, TMEM126B, glutamine, fatty acids, monocytes

## Abstract

Hypoxia poses a stress to cells and decreases mitochondrial respiration, in part by electron transport chain (ETC) complex reorganization. While metabolism under acute hypoxia is well characterized, alterations under chronic hypoxia largely remain unexplored. We followed oxygen consumption rates in THP-1 monocytes during acute (16 h) and chronic (72 h) hypoxia, compared to normoxia, to analyze the electron flows associated with glycolysis, glutamine, and fatty acid oxidation. Oxygen consumption under acute hypoxia predominantly demanded pyruvate, while under chronic hypoxia, fatty acid- and glutamine-oxidation dominated. Chronic hypoxia also elevated electron-transferring flavoproteins (ETF), and the knockdown of ETF–ubiquinone oxidoreductase lowered mitochondrial respiration under chronic hypoxia. Metabolomics revealed an increase in citrate under chronic hypoxia, which implied glutamine processing to α-ketoglutarate and citrate. Expression regulation of enzymes involved in this metabolic shunting corroborated this assumption. Moreover, the expression of acetyl-CoA carboxylase 1 increased, thus pointing to fatty acid synthesis under chronic hypoxia. Cells lacking complex I, which experienced a markedly impaired respiration under normoxia, also shifted their metabolism to fatty acid-dependent synthesis and usage. Taken together, we provide evidence that chronic hypoxia fuels the ETC via ETFs, increasing fatty acid production and consumption via the glutamine-citrate-fatty acid axis.

## 1. Introduction

Hypoxia arises when oxygen demands exceed its supply, which often is linked to diseases such as diabetes, autoimmune disorders, or cancer [1,2,3,4]. However, the severity of hypoxia varies, and its duration ranges from acute to chronic. In contrast to acute hypoxia, the term chronic hypoxia is not well defined, nor have its metabolic adaptations been explored. Previously, we specified chronic hypoxia as the phase of the hypoxic response where the protein expression of hypoxia inducible factors (HIF)-1α and HIF-2α decreased from their initial peaks under acute hypoxia to a steady state level slightly above normoxia [5]. This was accompanied by the corresponding regulation of HIF-inducible genes. They strongly increased under acute hypoxia, and decreased during over 48–72 h of oxygen shortage. Based on these experiments 1% oxygen and 72 h-incubations were chosen to study chronic hypoxia.

Mitochondria are major consumers of oxygen in cells, and they are forced to adapt to low oxygen availability. The activity of the respiratory chain substantially decreases, which is not only caused by a reduced mitochondrial mass, but it also results from adaptations within the individual respiratory chain complexes. For example, HIF-1 alters the composition of complex IV by increasing cytochrome c oxidase subunit (COX) 4-2 expression, which optimizes complex IV for oxygen usage under hypoxic conditions [6]. In contrast, the activity of complex I is reduced by a HIF-1-facilitated increase of NADH dehydrogenase (ubiquinone) 1 alpha subcomplex subunit 4-like 2 (NDUFA4L2) [7]. The adaptive response to chronic hypoxia also includes the proteasomal degradation of the complex I assembly factor TMEM126B. Thereby, the protein level of complex I is reduced, and oxygen consumption at this site of the ETC is impaired [8]. However, despite a substantial reduction of complex I amount and activity, the ETC continues to work at a lower level. This raises the question of how the ETC is fueled with electrons under acute vs. chronic hypoxia. Electrons can be delivered to the ETC by complex I (NADH:ubiquinone oxidoreductase), complex II (succinate dehydrogenase), electron-transferring flavoproteins (ETF), and the glycerin-3-phosphate shuttle. Each of these donors reduces ubiquinone to ubiquinol, which then passes electrons to complex III. Of note, different metabolites and metabolic pathways are involved in facilitating the distinct transfer mechanisms. Complex I takes over electrons from NADH, complex II oxidizes succinate to fumarate, and ETFs transfer reducing equivalents from acyl-CoA dehydrogenases [9,10]. ETFs are heterodimeric, FAD-containing proteins, found in all kingdoms of life. The primary function of ETFs is to carry one or two electrons from about 10 different flavoprotein-containing dehydrogenases to the respiratory chain, by forming transient complexes with their interaction partners [11,12]. ETFs transfer electrons to the membrane bound ETF-ubiquinone oxidoreductase (ETFDH) and from there, to the mitochondrial ubiquinone pool [13]. Mutations in those proteins may cause severe symptoms, such as multiple acyl-CoA dehydrogenase dysfunctional disease (MADD), underscoring the importance of ETFs [14,15]. In essence, these considerations point to pivotal roles of the TCA cycle and β-oxidation in fueling the ETC, with three metabolic pathways of major importance. Glycolysis produces pyruvate, which enters the Krebs cycle as acetyl-CoA. Glutamine, via glutamate and glutamate dehydrogenase, is converted into α-ketoglutarate to maintain the TCA cycle, while fatty acid oxidation contributes to ETF-mediated electron transfer. Interestingly, under hypoxia, fatty acid oxidation and the TCA cycle intermediate citrate are closely connected. The reductive carboxylation of α-ketoglutarate produces citrate, which leaves mitochondria to be cleaved by ATP citrate lyase (ACLY), with the resultant acetyl-CoA being consumed for fatty acid synthesis [16].

This study describes how electrons are channeled into the ETC under acute vs. chronic hypoxia. With the transition from acute to chronic hypoxia, cellular respiration shifted from being pyruvate-centered, to fatty acid- and ETF-based electron flow, in parallel with enhanced glutamine oxidation. A metabolic shift towards fatty acid oxidation was also noticed under conditions of impaired complex I activity, showing the dynamic range of adjusting oxygen-dependent respiration under hypoxia.

## 2. Materials and Methods

### 2.1. Cell Culture

THP-1 cells (derived from a male donor) were purchased from ATCC and incubated at 37 °C with 5% CO_2_ in RPMI medium (GE Healthcare, Munich, Germany), with the addition of 10% FCS, 1% pyruvate, 1% glutamine, and 1% penicillin/streptomycin (PAA Laboratories, Cölbe, Germany). ETFDH knockdown was induced by transfecting 2 × 10^6^ THP-1 cells with 50 nM small interfering RNAs (siRNAs) (ON-TARGETplus SMART pool, human ETFDH, Thermo Scientific, Karlsruhe, Germany), using Hiperfect (Qiagen, Hilden, Germany). For clustered regulatory interspaced short palindromic repeats (CRISPR)-mediated knockout of TMEM126B, THP-1 cells, stably transduced with a lentiviral vector containing Cas9 (pLentiCas9-Blast; Addgene #52962) and selected with 50 µg/mL blasticidine for 10 days, were again transduced with a lentivirus expressing the guide RNA against TMEM126B (pLentiCRISPRv2 ΔCas9 (derivate of pLentiCRISPRv2; Addgene #52961)). Afterwards, single-cell clones were created. For the experiments, five clones were selected, which showed knockout at the protein level. For the control, a non-target guide RNA (sgC) was used. For the metabolic studies, MDA-MB-231 cells (ATCC) were cultured in Dulbecco’s Modified Eagle’s Medium (DMEM) medium (GE Healthcare), with the addition of 10% fetal calve serum (FCS), 1% glutamine, and 1% penicillin/streptomycin (PAA Laboratories, Cölbe, Germany).

### 2.2. Hypoxic Incubation

Hypoxic incubations were performed in a SciTive Workstation (Baker Ruskinn, Leeds, UK) at 1% O_2_ and 5% CO_2_ for the times indicated. To ensure constant nutrient availability, equilibrated fresh media was added to the cells after 24 h and 48 h.

### 2.3. Western Analysis

Cells were lysed in a buffer containing 4% sodium dodecyl sulfate (SDS), 150 mM NaCl, and 100 mM Tris/HCl, pH 7.4, and sonicated. Protein content was determined by a protein assay kit (Bio-Rad, Munich, Germany), and 100 µg protein was loaded onto a 10% SDS gel. Gels were blotted by using a Trans Blot Turbo blotting system (Bio-Rad).

Membranes were blocked in 5% milk in Tris-buffered saline with 0.05% tween 20 (TBS-T) for tubulin (Sigma-Aldrich, Munich, Germany), or 5% bovine serum albumin (BSA) in TBS-T for TMEM126B (Atlas Antibodies via Sigma-Aldrich, Munich, Germany), electron transferring flavoprotein-ubiquinone oxidoreductase (ETFDH) (Abcam, Berlin, Germany), and oxidative phosphorylation (OXPHOS) Western cocktail (abcam). Enhanced chemiluminescence on a C-DIGIT scanner (Licor, Lincoln, USA), or fluorescence on an Odyssey scanner (Licor), were quantified with Image Studio Digits 5.0 (Licor).

### 2.4. Real-Time PCR

RNA was isolated using peqGold (Peqlab, Erlangen, Germany) and measured by using a Nanodrop ND-1000 spectrophotometer (Peqlab, Erlangen, Germany). Reverse transcription was performed with the Maxima First Strand cDNA Synthesis Kit for RT-PCR (Thermo Fisher Scientific, Waltham, USA). RNA expression was analyzed by using a SYBR green fluorescent mix (Thermo Fischer Scientific) on a CFX96 Real Time PCR Detection System (Bio-Rad), and normalized to TATA box binding protein (TBP). All primer sequences are listed in Table 1.

### 2.5. Seahorse

The cellular oxygen consumption rate (OCR) and the extracellular acidification rate (ECAR) were analyzed using a Seahorse 96 extracellular flux analyzer (Agilent, Waldbronn, Germany). THP-1 cells were plated in Seahorse 96-well cell culture plates one day prior to the measurements, and equilibrated for 30 min before recordings were made in Krebs Henseleit buffer (111 mM NaCl, 4.7 mM KCl, 1.25 mM CaCl_2_, 2 mM MgSO_4_, 1.2 mM Na_2_HPO_4_) supplemented with 5 mM l-glucose and 1 mM l-glutamine. Cells were treated with 1 μM rotenone (Sigma-Aldrich, Munich, Germany), 50 µM etomoxir (Cayman Chemicals), 30 µM Bis-2-(5-phenylacetamido-1,3,4-thiadiazol-2-yl)ethyl sulfide (BPTES, Sigma-Aldrich, Munich, Germany), 20 µM UK5099 (Sigma-Aldrich, Munich, Germany), or 1 µM atpenin A5 (Cayman Chemicals).

### 2.6. Mitochondrial Membrane Potential

Cells were incubated with 1 µM of the mitochondrial membrane potential-sensitive dye JC-1 (Thermo Fischer Scientific) for 20 min at 37 °C, under hypoxia. Afterwards, cells were washed with PBS and green (fluorescein, FITC) vs. red (PE 561-A) fluorescence was measured on an LSRFortessa (BD, Heidelberg, Germany).

### 2.7. Quantification of Free Amino Acids in Cells and Cell Culture Media

Exactly 25 μL sample volumes were used for amino acid analysis. Sample preparation was performed by using the EZ:faast LC-MS free amino acid analysis kit (Phenomenex, Aschaffenburg, Germany) according to the manufacturer’s instructions, with minor modifications. Only 10 µL of the internal standard mix were applied to all samples and to the standard curve. After processing, the samples and standards were evaporated and resolved in 75 µL in 66.6% methanol containing 10 mM ammonium formate. Analysis of metabolites was performed by LC-MS/MS, using the EZ:faast AAA-MS HPLC column on an Agilent 1290 Infinity LC system (Agilent) coupled to a QTrap 5500 mass spectrometer (Sciex, Darmstadt, Germany). The column temperature was set to 35 °C. Gradient elution was performed with 10 mM ammonium formate in water (mobile phase A) and 10 mM ammonium formate in methanol (mobile phase B). Conditions for the separation were a 13 min gradient from 68% B to 83% B, followed by an equilibration step. The flow rate was set to 250 µL/min. The injection volume was 1 µL. Electro spray ionization in positive mode was employed. The ion source parameter was as follows, CUR 25 psi, IS 4000 °C, TEM 425 °C, GS1 40 psi, GS2 40 psi. Calibration curves were performed with the authentic standards from the EZ:faast kit. The intensities of the measured metabolite were normalized to internal standards. Analyst 1.6.2 and MultiQuant 3.0 (Sciex, Darmstadt, Germany), were used for data acquisition and analysis, respectively.

### 2.8. Quantification of TCA Cycle Metabolites

The cell homogenate (150 µL) or cell culture medium (100 µL) were mixed with 50 µL or 25 µL isotope-labeled internal standard, respectively. Samples were evaporated in a vacuum concentrator (Eppendorf, Hamburg, Germany) at 30 °C, resolved in 50 µL water, and subsequently transferred to the LC-MS system. Cell culture medium samples were directly transferred to the LC-MS system.

Liquid chromatography mass spectrometry was performed on an Agilent 1290 Infinity LC system (Agilent) coupled to a QTrap 5500 mass spectrometer (Sciex, Darmstadt, Germany). The reversed-phase LC separation was performed using a Waters Acquity UPLC HSS T3 column (150 mm × 2.1 mm, 1.8 µm (Waters, Eschborn, Germany)) at 40 °C. Gradient elution was performed with 0.15% formic acid in water (mobile phase A) and 0.15% formic acid in acetonitrile (mobile phase B) at a flow rate of 400 µL/min. Gradient conditions were 2% B for 1.5 min, followed by a 3 min gradient to 100% B, followed by a cleaning and equilibration step, with 10 min of total LC run time. The injection volume was 2.5 μL for all samples. The autosampler temperature was 6 °C. Electrospray ionization at 400 °C, with 4500 V in negative ionization mode, was employed. The ion source gas parameters were as follows, CUR 30 psi, GS1 45 psi, GS2 25 psi. The specific MRM transition for every compound was normalized to the appropriate isotope-labeled internal standard. Calibration curves were performed with authentic standards. Analyst 1.6.2 and MultiQuant 3.0 (Sciex, Darmstadt, Germany), were used for data acquisition and analysis, respectively.

### 2.9. Statistics

Data are expressed as mean values ± SEM. Statistically significant differences were calculated after analysis of variance (ANOVA) and Bonferroni’s test or Students *t*-test; *p* < 0.05 was considered as significant.

## 3. Results

### 3.1. A Metabolic Phenotype Change in THP-1 Cells Under Hypoxia

**Figure 1:**
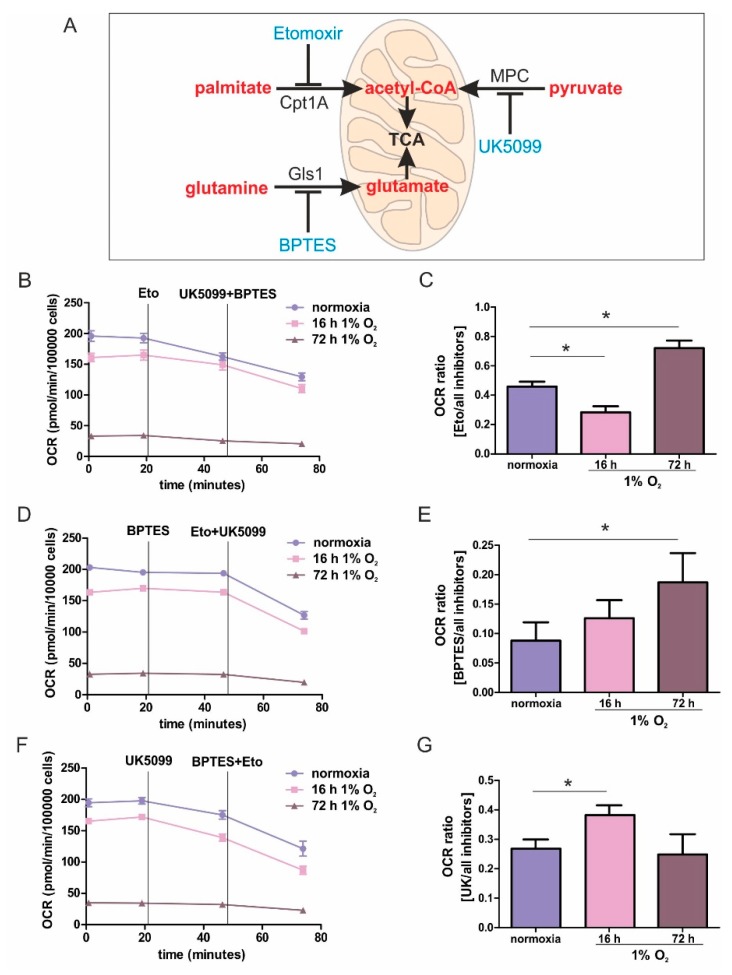
Mitochondrial substrate fuel under normoxia, and acute and chronic hypoxia. (**A**) Scheme of the mitochondrial utilization of palmitate by carnitine *O*-palmitoyltransferase 1 (Cpt1A), pyruvate by the mitochondrial pyruvate carrier (MPC), and glutamine by glutaminase 1 (Gls1), with corresponding inhibitors (blue). (**B**–**G**) THP-1 monocytes were incubated for 16 or 72 h under 1% O_2_ vs. normoxia. The oxygen consumption rate (OCR) was measured using a Seahorse flux analyzer. Fatty acid uptake was inhibited by etomoxir (eto), pyruvate import was antagonized by UK5099, while glutamate synthesis was suppressed by Bis-2-(5-phenylacetamido-1,3,4-thiadiazol-2-yl)ethyl sulfide (BPTES). (**B**,**D**,**F**) Fitted, representative OCR curves connecting rates of individual OCR rates, with and without inhibitors, are shown by straight lines. Exemplified original traces are shown in supplementary Figure S1B–D. (**C**,**E**,**G**) OCR is calculated as the ratio of oxygen consumption seen with one inhibitor, compared to all inhibitors. Data are mean values ± SEM, *n* = 3, * *p* < 0.05.

To explore the metabolic pathways that fuel the ETC under acute and chronic hypoxia, a Seahorse flux analyzer was used to follow oxygen consumption in THP-1 monocytes, depending on pyruvate, glutamine, or fatty acid ingestion (Figure 1A). Cells were incubated for 16 h (acute hypoxia) or 72 h (chronic hypoxia) at 1% O_2_, compared to normoxic controls. These time points were established in previous studies to reflect conditions of acute vs. chronic hypoxia [5,8]. Measurements were performed in Krebs Henseleit buffer supplemented with glutamine and glucose.

The dependency on a distinct substrate pathway was expressed as the ratio of interference with one pathway, compared to blocking all pathways. The experimental protocol and data acquisition are illustrated in Figure S1. In general, cellular respiration was slightly reduced following incubations under acute hypoxia for 16 h, compared to normoxia, which became more pronounced with chronic hypoxic pre-treatments for 72 h (Figure 1B,D,F). However, despite a prominently reduced respiration under chronic hypoxia, a residual respiration of roughly 50 pmol/min/100,000 cells remained. To capture oxygen consumption rates (OCR) demanding fatty acids, we used etomoxir to block carnitine *O*-palmitoyltransferase 1 (Cpt1A), which imports long chain fatty acids into mitochondria. Following OCR for roughly 30 min, we added UK5099 to block the mitochondrial uptake of pyruvate and BPTES to inhibit glutaminase 1 (Gls1) (Figure 1B and Figure S2A). Etomoxir, compared to the combination UK5099/BPTES generated an OCR ratio of roughly 0.5, pointing to a balanced use of electrons from fatty acid oxidation vs. glutamate and pyruvate oxidation. An acute hypoxic challenge decreased the ratio to a value of around 0.3, implying that inhibition by UK5099/BPTES became more pronounced, while following chronic hypoxia, the ratio increased to roughly 0.7, suggesting a more dominant role of fatty acid oxidation (Figure 1C and Figure S2B). To analyze the OCR-dependency on glutamine, Gls1 was inhibited using BPTES, followed by the addition of UK5099/etomoxir (Figure 1D and Figure S2C). The reliance on glutamine oxidation was modest under all conditions, which was reflected by a low ratio of 0.1 to 0.2. However, glutamine oxidation became more pronounced by the addition of UK5099/etomoxir (Figure 1E). After acute hypoxia, cells were slightly more dependent on glutamine oxidation than normoxic cells, with this dependency becoming significant following chronic hypoxic incubations. The role of pyruvate oxidation was analyzed by using UK5099 to interfere with the mitochondrial pyruvate carrier (MPC), followed by the addition of etomoxir/BPTES (Figure 1F). Under normoxia, the ratio of 0.25 implied a moderate occurrence of pyruvate oxidation (Figure 1G). After acute hypoxia, the ratio unexpectedly increased to values of around 0.4, pointing to the importance of pyruvate oxidation in monocytic cells under acute hypoxia. When hypoxia became chronic, pyruvate consumption decreased to values of normoxic incubations.

These results established pronounced differences in substrate usage underlying OCR, comparing acute vs. chronic hypoxia. Under prolonged periods of oxygen shortage, the dependence on glutamate and fatty acid oxidation was striking. This provoked two questions: First, how does β-oxidation fuel the respiratory chain under chronic hypoxia, and second, does glutamine enhance electron transfer via complex II, or is it processed to citrate, to enhance fatty acid synthesis followed by their β-oxidative destruction.

### 3.2. ETFs are Important for Allowing Oxygen Consumption under Chronic Hypoxia

**Figure 2:**
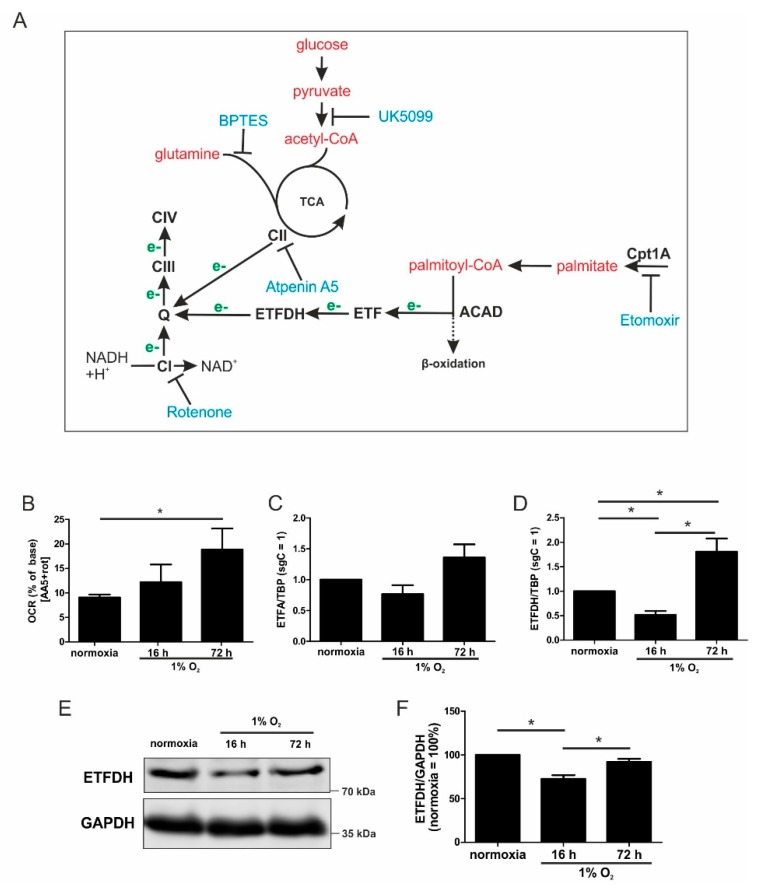
ETFs fuel ETC under chronic hypoxia. (**A**) Scheme of metabolic intermediates (red) that provide electrons (e-, green) to the electron transport chain complexes (CI, III, and IV), including the inhibitors (blue). Electrons are transferred to ubiquinone (Q) either from the tricarboxylic acid cycle (TCA) via complex II (CII), or via fatty acids imported into the mitochondria by carnitine *O*-palmitoyltransferase 1 (Cpt1A) using acyl-CoA dehydrogenases (ACAD) and electron-transferring flavoproteins (ETF). (**B**) THP-1 cells were incubated for 16 vs. 72 h under hypoxia, and OCR was measured in the presence of atpenin A5 (AA5) and rotenone (rot). The basal respiration was set to 100%. (**C**) ETFA mRNA expression was analyzed in hypoxic THP-1 cells and normalized to TBP (*n* = 7). (**D**) ETFDH mRNA expression, normalized to the TATA box binding protein (TBP), was followed in cells incubated for 16 vs. 72 h under hypoxia (*n* = 7). (**E**) Western analysis of ETFDH and GAPDH at the indicated times of hypoxia. (**F**) Quantification of E (*n* = 4). Data are mean values ± SEM, * *p* < 0.05.

The respiratory chain receives electrons via complex I, complex II, and ETFs. Figure 2A provides an overview of these pathways, also indicating sites of interference by various pharmacological agents. Based on the findings that fatty acid oxidation gains importance over other pathways under chronic hypoxia to provide electrons for oxygen reduction, the ETF-system was focused upon. To prove the importance of ETF-mediated electron transfer under chronic hypoxia, complex I was inhibited with rotenone (rot), and complex II activity was compromised by atpenin A5 (AA5). Thereby, the ETFs remained as possible electron carriers (Figure 2B). As expected, residual respiration following complex I and complex II inhibition was significantly higher after chronic hypoxia, compared to normoxia or acute hypoxia. Then, the hypoxic mRNA expression of ETFA and ETFDH was analyzed (Figure 2C,D). Their expression decreased under acute hypoxia, but this increased under chronic conditions, with changes of ETFDH reaching significance. Next, the expression of ETFDH was verified by Western analysis, including quantification (Figure 2E,F). There was a reduction under acute hypoxia, and a comparable relative increase under chronic hypoxia. These results may underscore the importance of ETFs in fueling ETC under chronic hypoxia.

### 3.3. An ETFDH Knockdown Decreased Respiration and the Mitochondrial Membrane Potential

**Figure 3:**
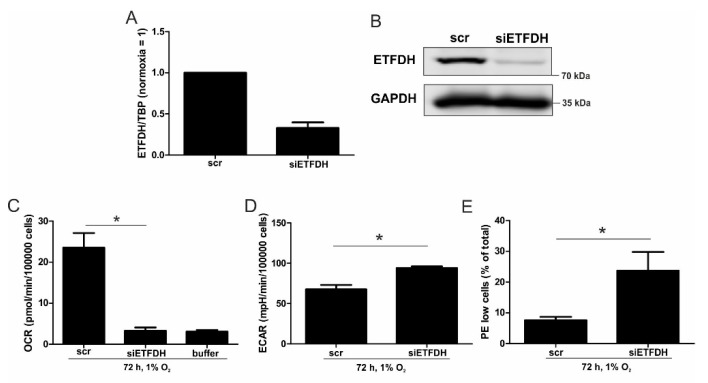
OCR with a knockdown of ETFDH. (**A**) THP-1 cells were transfected with siRNA against ETFDH (siETFDH) or a scrambled control (scr). mRNA expression of ETFDH was analyzed after three days and normalized to TBP. (**B**) ETFDH protein was analyzed by Western analysis, with GAPDH serving as a loading control. (**C**) OCR of chronic hypoxic scr and siETFDH cells were analyzed. The buffer served as a negative control for non-cellular OCR. (**D**) The extracellular acidification rates (ECAR) of chronic hypoxic scr and siETFDH cells were measured by a Seahorse flux analyzer. (**E**) Scr and siETFDH cells were incubated for 72 h under hypoxia, stained with the mitochondrial dye JC-1, and measured by fluorescence activated cell sorting (FACS). The graph shows the percentage of cells with a low mitochondrial membrane potential (PE-low and FITC-high) under chronic hypoxia (*n* = 4). Data are mean values ± SEM, * *p* < 0.05.

To further characterize how ETFs contribute to residual respiration under chronic hypoxia, a siRNA-mediated knockdown of ETFDH (siETFDH) in THP-1 cells was generated and compared to a scrambled control (scr) (Figure 3). Knockdown efficacy at the mRNA level was roughly 70% (Figure 3A).

A reduced protein amount was corroborated three days after inducing knockdown, as seen from Western analysis (Figure 3B). Subsequently, oxygen consumption was measured in scrambled- and siETFDH-transfected cells when incubated under hypoxia for 72 h (Figure 3C and Figure S2D). Oxygen consumption markedly declined when ETFDH was missing, with values as low as the buffer control. As a potential compensatory mechanism, the rate of extracellular acidification increased in these cells under all conditions (Figure 3D and Figure S2E). Thus, ETFs appear to maintain electron flow through the respiratory chain under chronic hypoxia, which is a prerequisite for preserving the mitochondrial membrane potential (ΔΨm) and consequently, healthy mitochondria. To assess the impact of ETFs on ΔΨm under chronic hypoxia, cells were transfected with siRNAs against ETFDH, and incubated for 72 h under hypoxia. Afterwards, cells were stained with 1 µM JC-1, a dye, which is taken up by mitochondria, and emits ΔΨm-sensitive fluorescence (Figure 3E and Figure S2F). In control transfected cells, about 8% of the cells showed a decreased ΔΨm, which was associated with a low red fluorescence. In cells with a knockdown of ETFDH, this number increased significantly, to about 23%. Taking a knockdown efficiency of roughly 70% into account, these data indicate that ETFs are important for maintaining ΔΨm, and thus, to preserving mitochondrial integrity under chronic hypoxia.

### 3.4. Glutamine Metabolism

**Figure 4:**
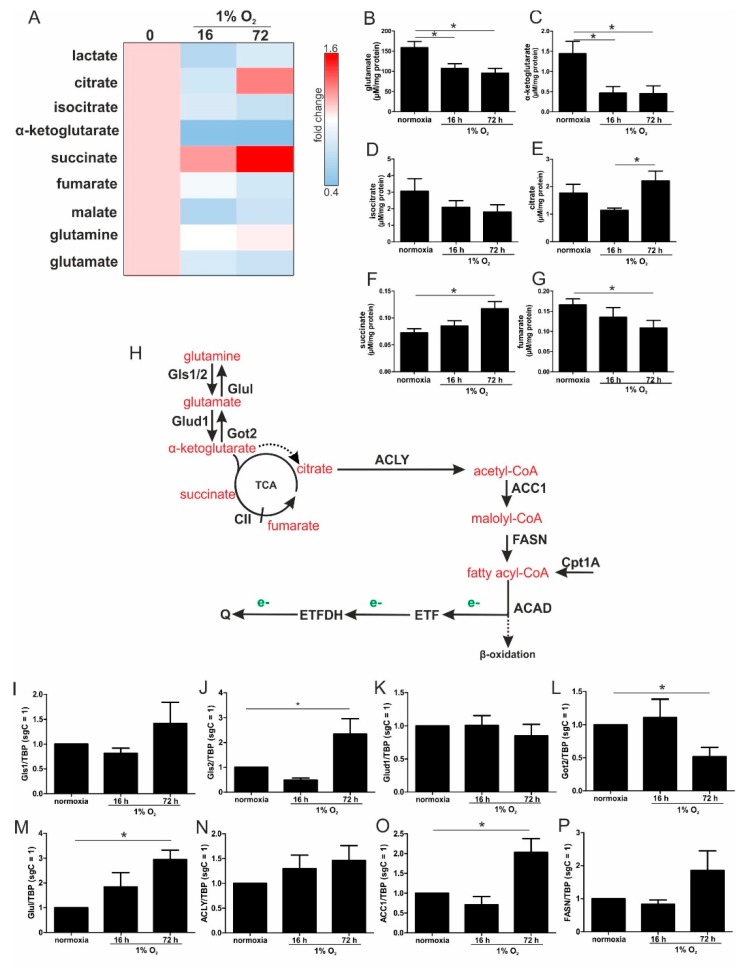
Pathways of glutamine metabolism. (**A**) MDA-MB-231 cells were incubated for the indicated time points under hypoxia, and metabolites were measured by mass spectrometry, followed by heatmap visualization. (**B**–**G**) Metabolite concentrations determined under acute and chronic hypoxia. (**H**) Scheme linking glutamine processing by glutaminase (Gls), glutamate dehydrogenase (Glud1), glutamine synthetase (Glul), and aspartate aminotransferase (Got2) to fatty acid metabolism via ATP-citrate lyase (ACLY), acetyl-CoA carboxylase 1 (ACC1) fatty acid synthase (FASN), carnitine *O*-palmitoyltransferase 1 (Cpt1A), and acyl-CoA dehydrogenase (ACAD) which transfers electrons (e-, green) to ubiquinone (Q) via electron-transferring flavoproteins (ETF). The dashed arrow indicates the conversion of α-ketoglutarate to citrate under hypoxic conditions. (**I**–**P**) THP-1 cells were incubated for 16 vs. 72 h under hypoxia. mRNA expression of Gls1 (**I**), Gls2 (**J**), Glud1 (**K**), Got2 (**L**), Glul (**M**), ACLY (**N**), ACC1 (**O**), and FASN (**P**) was analyzed and normalized to TBP (*n* = 7). Data are mean values ± SEM, * *p* < 0.05.

During the transition from acute to chronic hypoxia, not only did fatty acid oxidation gain importance, but also glutamine metabolism. Therefore, we analyzed metabolites that were potentially derived from glutamine, with relevance for the TCA cycle in normoxia vs. acute and chronic hypoxia (Figure 4A). The analyses were performed in MD-MB-231 cells, since adherent cancer cells are an established model to follow metabolic changes under hypoxia, which has not been verified for monocytes so far.

Levels of glutamate, α-ketoglutarate, and isocitrate decreased after 16 and 72 h hypoxia (Figure 4B–D). Citrate significantly increased under chronic hypoxia (Figure 4E), which was also seen for succinate (Figure 4F). In contrast, fumarate was reduced (Figure 4G), which suggests a diminished degree of complex II (CII, SDH) activity under chronic hypoxic conditions. Previously, it was noticed that α-ketoglutarate is converted to citrate under hypoxic conditions [16]. The decrease in α-ketoglutarate may indicate that it is metabolized in part to succinate and/or isocitrate, which in turn accumulates as citrate, which then can be further processed for fatty acids. To substantiate these considerations, we switched back to THP-1 cells and analyzed the mRNA levels of distinct enzymes that may account for such alterations.

### 3.5. Chronic Hypoxia Increased Glutamine and Fatty Acid Metabolism

Since glutamine may contribute to fatty acid synthesis, the mRNA expression of crucial enzymes involved in glutamine metabolism were analyzed (Figure 4H). Glutaminase (Gls) 1 and Gls2 remained at control level under acute hypoxia, but both were expressed under chronic hypoxic conditions, with only Gls2 reaching significance (Figure 4I,J). Following the conversion of glutamine to glutamate, the latter was further processed to α-ketoglutarate by glutamate dehydrogenase 1 (Glud1), which remained unaltered under hypoxia (Figure 4K). Along those lines, the expression of aspartate aminotransferase (Got2), which catalyzes the formation of glutamate from α-ketoglutarate, decreased under chronic hypoxia, while glutamine synthetase (Glul) slightly increased under acute hypoxia, which reached significance under chronic conditions (Figure 4L,M). Since the mRNA expression of enzymes involved in α-ketoglutarate synthesis were increased under chronic hypoxia, which is paralleled by an increase in citrate, the question arose as to whether citrate might be used for fatty acid synthesis. Therefore, mRNAs of ATP–citrate lyase (ACLY), acetyl-CoA carboxylase 1 (ACC1), and fatty acid synthase (FASN) were analyzed (Figure 4N–P). While ACLY and FASN were elevated under chronic hypoxia without reaching significance, mRNA expression of the rate limiting enzyme ACC1 was significantly increased. These considerations may suggest that glutamine is used to produce α-ketoglutarate, with a further transformation to citrate. Citrate can be used for the synthesis of fatty acids, with their breakdown resulting in electron delivery for the ETC, and then channeled into the respiratory chain via ETFs.

### 3.6. Complex I Abundance and Oxygen Consumption are Reduced in sg126B Cells

**Figure 5:**
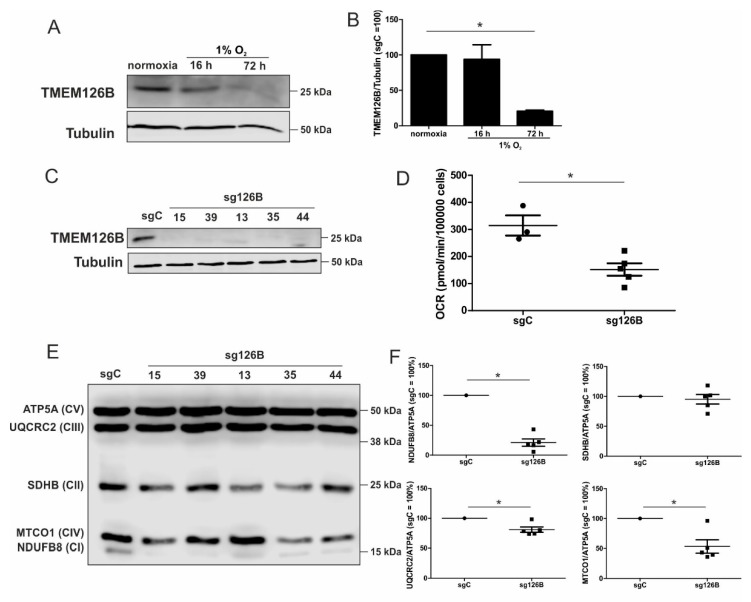
Characterization of a TMEM126B knockout. (**A**) Western analysis of TMEM126B vs. tubulin, under acute and chronic hypoxia. (**B**) Quantification of (A) (*n* = 3). (**C**) Knockout THP-1 cells were generated via CRISPR/Cas9 gene editing using a single guide RNA against TMEM126B (sg126B) or non-target control (sgC). Western analyses of the indicated clones were performed to validate the knockout. (**D**) OCR was measured in sg126B clones compared to sgC controls. (**E**) Western analyses of respiratory chain complexes in sgC and sg126B cells. (**F**) Quantification of (E). Mean values of four independent experiments are depicted in the graph, * *p* < 0.05.

Previous studies showed that the mitochondrial complex I assembly factor TMEM126B is proteolytically degraded under chronic hypoxia and consequently, the abundance and activity of complex I were decreased [8]. Here, these findings were corroborated in monocytic THP-1 cells (Figure 5A,B). This provoked the question of whether cells lacking TMEM126B adapt under ambient oxygen partial pressure in the ways described for chronic hypoxic cells.

To mimic the TMEM126B-induced phenotype and thus, a certain aspect of chronic hypoxia under normoxia, we created a knockout of TMEM126B (sg126B), and a corresponding control (sgC) in THP-1 cells, using the CRISPR-Cas9 technology. Since a pool of transduced cells showed residual TMEM126B expression in Western analysis, single cell clones were created. Analysis of TMEM126B expression in clones 15, 39, 13, 35, and 44 revealed a nearly-complete to complete loss of protein (Figure 5C). As anticipated, basal respiration of all knockout clones was markedly decreased (Figure 5D). As depicted in Figure 5E,F, we verified that the knockout of TMEM126B indeed attenuated complex I abundance, according to Western analysis of NDUFB8 (complex I). Furthermore, MTCO1 (complex IV), SDHB (complex II, SDH), UQCRC2 (complex III), and ATP5A (complex V) were analyzed (Figure 5F). A reduced abundance of complex I and a severely compromised OCR underscored the notion that a knockout of TMEM126B replicated conditions of chronic hypoxia, although OCR was roughly two to three times higher compared to the OCR observed under chronic hypoxia.

### 3.7. OCR in sg126B Cells Demands Fatty Acid Oxidation

**Figure 6:**
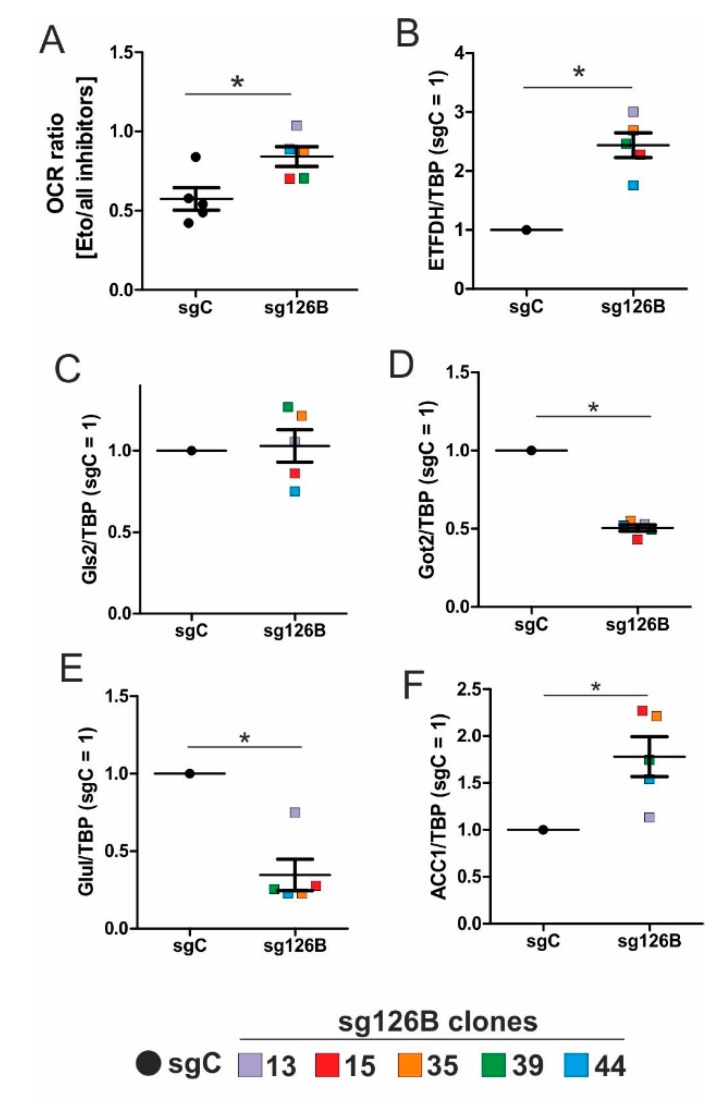
Metabolic alterations in TMEM knockout (sg126B) clones. (**A**) The Ratio of OCR in sgC and sg126B clones with the addition of etomoxir, and subsequent supplementation of UK5099, as well as BPTES. (**B**–**F**) mRNA analyses of ETFDH (**B**), Gls2 (**C**), Got2 (**D**), Glul (**E**), and ACC1 (**F**) in sgC and sg126B cells normalized to TBP. Mean values of five independent experiments for each clone are depicted in the graph, * *p* < 0.05. For individual expression profiles, see Figure S3.

The ratio of the OCR under etomoxir treatment, compared to the addition of all inhibitors (etomoxir, UK5099, BPTES) increased in TMEM126B knockout cells, compared to the non-targeting controls (Figure 6A). An increased OCR ratio during Cpt1 inhibition, compared to all inhibitors, implies that the respiratory chain is predominantly fueled by fatty acids in cells when complex I is impaired (as seen in sg126B cells). Rates of oxygen consumption were analyzed in all five clones, and each clone is represented by a different color.

The mRNA expression of ETFDH increased in sg126B clones, which parallels the observations seen under chronic hypoxia (Figure 6B vs. Figure 2D). Conclusively, metabolic adaptations occurring in TMEM126B knockout cells reflect the conditions occurring under chronic hypoxia. The remaining low rates of oxygen consumption (Figure 5D) and the residual complex I expression (Figure 5F) may indicate/guarantee the operation of this fuel pathway at a low but persistent rate.

### 3.8. Increased Rates of Glutamine and Fatty Acid Metabolism in sg126B Cells

By analogy to chronic hypoxia, genes involved in glutamine metabolism were analyzed in sg126B clones. Independent experiments were performed in each clone, with the mean values being depicted in the graphs. Information on mRNA expression in each individual clone can be found in Supplementary Figure S2. While Gls2 was not regulated, Got2 and Glul were significantly decreased (Figure 6C–E), supporting the idea that glutamine metabolism is shifted towards α-ketoglutarate. With relevance to fatty acid synthesis, ACC1 mRNA expression was increased in the sg126B clones (Figure 6F). Of interest, ACC1, Got2, and ETFDH expression were significantly regulated as seen under chronic hypoxia. Therefore, it seems advisable to conclude that metabolic alterations seen under chronic hypoxia are mimicked by a genetic deletion of complex I, which is associated with a drastically reduction in basal oxygen consumption.

## 4. Discussion

While metabolic adaptations under acute hypoxia are reasonably well-studied, the metabolic flexibility during long-term oxygen deprivation was less explored. Here, we examined the metabolic changes in THP-1 monocytes during the transition from acute to chronic hypoxia. With prolonged periods of hypoxia, cells show an increased demand for the oxidization of fatty acids to channel electrons via ETFs into the respiratory chain. Cellular respiration demands oxygen, and logically, it is impaired under hypoxia. It is generally accepted that pyruvate dehydrogenase is impaired due to phosphorylation via PDK1 (pyruvate dehydrogenase kinase 1), which itself is a hypoxia inducible gene. Therefore, the TCA cycle should be slowed down, and the generation of redox equivalents for complex I are reduced. With a slower turnover of the TCA cycle, succinate dehydrogenase also may face reduced substrate availability, and the delivery of electrons via complex II may be impaired. Along these lines, fatty acids are stored in lipid droplets, making them inaccessible for mitochondrial β-oxidation. Consequently, channeling electrons via ETFs into the ETC is attenuated. Besides the generation of reducing equivalents, components of the respiratory chain themselves are also subjected to hypoxic regulation. However, in this case, conflicting concepts exist. On one hand a decreased activity of complex I had been noticed, while on the other hand, changes in the complex formation of cytochrome c oxidase may occur, to maximize the limited usage of oxygen. Even if respiration under acute hypoxia is reduced, cells continue to maintain some mitochondrial respiratory activity, as this seems important for cancer cells to survive and to metastasize. The situation even becomes more blurry if we move from acute to chronic hypoxia. Under chronic hypoxia, expression of beta-transducin repeat-containing protein 1 (β-TrCP1) is upregulated, which ubiquitinates and destroys the complex I assembly factor TMEM126B. Consequently, complex abundance is drastically lowered, and its activity is severely impaired. Compared with acute hypoxia showing an OCR of roughly 150 pmol/min/100,000 cells, oxygen consumption under chronic hypoxia drops to values of below 50 pmol/min/100,000 cells. Addressing mitochondrial fuel usage under chronic hypoxia, we assessed the flexibility of THP-1 cells in oxidizing three critical mitochondrial fuels, such as glucose, glutamine, or long-chain fatty acids. With a Seahorse flux analyzer, we followed the rates of substrate oxidation of each fuel, by measuring mitochondrial respiration. Therefore, we initially inhibited one pathway, followed by blocking the remaining two. Using a combination of etomoxir, which interferes with the transport of fatty acids into mitochondria, UK5099, which blocks the transport of pyruvate into mitochondria, and BPTES, which inhibits the conversion of glutamine to glutamate, the dependency of cells on different substrates can be explored. Expressing the ratio of OCR with one inhibitor vs. all inhibitors allows a standardized output value and the comparison of data.

For chronically hypoxic THP-1 monocytes, we noticed an unexpected demand for the oxidization of fatty acids and glutamine, while acutely hypoxic cells appeared to rely on pyruvate, with a decreased importance of fatty acid oxidation. Also, liver cells attenuated β-oxidation under acute hypoxia, mediated via HIF-2 [17,18]. Corroborating these findings, the Cpt1-facilitated mitochondrial uptake of fatty acids is impaired under hypoxia [19]. Pyruvate-dependent respiration under acute hypoxia is perplexing. However, monocytes, compared to fully differentiated macrophages, only show a modest upregulation of hypoxic genes [20,21]. The expression of glycolytic enzymes, as well as the secretion of lactate under hypoxia, was low compared to macrophages exposed to hypoxia, and they only reached values already found in differentiated monocytes, i.e., macrophages under normoxia. Taking the minor regulation of hypoxic genes in monocytes into consideration, it is no longer surprising that we do see a slight dependence on pyruvate under acute hypoxia.

During the progression from acute to chronic hypoxia, we noticed a decline of HIF-1α, HIF-2α, as well as HIF-target gene expression [5,22]. Reaching this new ‘set point’, which is apparently distinct from acute hypoxic responses, cells may reset their metabolism accordingly. Since electrons from β-oxidation are transferred via ETFs to ubiquinone, oxygen consumption was measured with the inhibition of complex I and complex II, leaving ETFs as a possible electron source. Under these conditions, we noticed an increase in respiration under chronic hypoxia compared to normoxia, pointing to the importance of ETFs. The ETF-dependency was proven by a siRNA-mediated knockdown of ETFDH, reducing respiration under chronic hypoxia to background levels. In addition, we observed a decrease in ΔΨm in siETFDH cells under chronic hypoxia. Apparently, the ETF system appears to be crucial for maintaining mitochondrial integrity, guaranteeing/preserving their crucial functions in metabolism (e.g., glutaminolysis and β-oxidation) and signal transduction (e.g., the formation of reactive oxygen species and inflammatory signaling). Looking for distinct metabolites, succinate accumulated under chronic hypoxia, while fumarate decreased, which implied a lower complex II activity. This constellation makes an electron transfer via ETFs in an exclusive way, to fuel ETC under chronic hypoxia, and it explains the increased dependency on fatty acid oxidation. Besides, we noticed an increasing demand for glutamine under chronic hypoxia. Also, levels of citrate increased under chronic hypoxia, and the mRNA expression of genes involved in glutamine breakdown pointed to the possibility that glutamine is processed to α-ketoglutarate and citrate. Citrate in turn can be used for the production and metabolism of fatty acid oxidation via ACLY, ACC1, and FASN [23]. Supporting our hypothesis of a metabolic switch to β-oxidation under chronic hypoxia, pulmonary artery endothelial cells expressed increased levels of FASN under chronic hypoxic conditions to produce palmitate, which facilitates increased proliferation [24]. In mesenchymal stem cells BNIP3 is needed under hypoxia to induce FASN, and to synthesize fatty acids [25]. Restricting electron flow into the ETC via ETFs under chronic hypoxia fits with the reduced availability of oxygen but leaves the mitochondria as the central organelles in metabolism intact. A therapeutic approach uses etomoxir to inhibit β-oxidation in tumors, and to enhance the impact of irradiation [26]. This approach especially targets hypoxic cells, which, according to our study, are highly dependent on β-oxidation. To explore whether the decrease of complex I under chronic hypoxia might be sufficient to induce this metabolic switch, the complex I assembly factor TMEM126B was depleted in THP-1 cells, which reduced complex I abundance and activity. Interestingly, sg1261B cells reflect several characteristics of chronic hypoxic cells. Of importance, these cells also show a strong dependency on fatty acid oxidation. β-oxidation might be a critical pathway to compensate for a severe complex I deficiency, and to maintain mitochondrial integrity. Normally, fatty acid synthesis and β-oxidation are reciprocally regulated, in order to avoid a futile cycle. Apparently this fundamental regulatory principle is overruled when cells adapt to stresses such as chronic hypoxia. The same phenomena of fatty acid synthesis simultaneously occurring alongside β-oxidation is reported for the adaptation to cold stress in brown adipose tissue [27], or under conditions of acidosis [28], pointing to unique metabolic profiles under potentially harmful cellular conditions. Corbet and coworkers reported an increase in acetylation during acidosis, which reduced mitochondrial complex I activity. In parallel, histone deacetylation resulted in a decline in ACC2 expression. This in turn allowed for the concomitance of fatty acid oxidation and synthesis via reductive carboxylation of glutamine-derived α-ketoglutarate, which subsequently supported complex I-independent oxidative phosphorylation. However, under our experimental conditions we ruled hypoxic-elicited acidosis out, as we frequently changed media, and moreover, we took the low capacity of monocytes to acidify the media into consideration. In addition, it might be of importance that ACC1, which was upregulated in our study, is cytosolic, and it is believed to be the primary acetyl-CoA carboxylase involved in de novo fatty-acid synthesis, while ACC2, studied by Corbet and coworkers, appears to regulate fatty-acid oxidation through the malonyl-CoA-mediated inhibition of Cpt1 [29]. Nevertheless, an increase in histone acetylation due to alterations in ACC expression, which may alter the transcriptional profiles of cells, cannot be ruled out, and this needs consideration in further studies.

**Figure 7:**
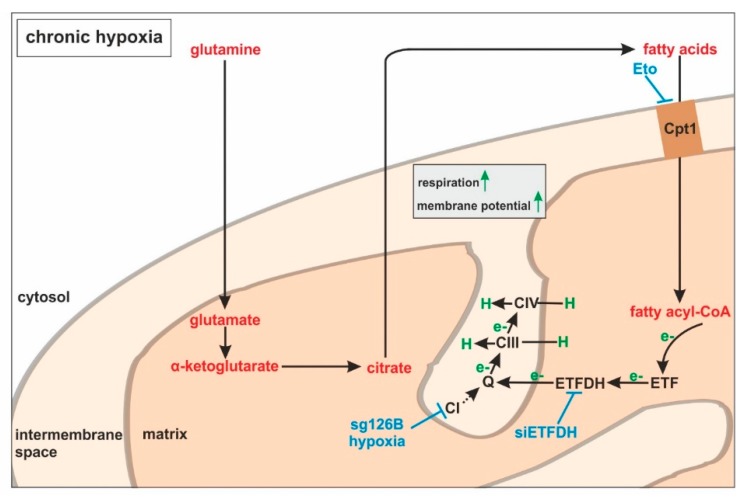
ETFs fuel the respiratory chain under chronic hypoxia. Under chronic hypoxia, glutamine is converted to glutamate, and further processed via α-ketoglutarate to citrate. In turn, citrate is used to produce fatty acids, which are imported into the mitochondria by carnitine *O*-palmitoyltransferase 1 (Cpt1). During fatty acid catabolism, electrons (e−) are transferred to electron-transferring flavoproteins (ETFs) and ETF−ubiquinone oxidoreductase (ETFDH). ETFDH channels electrons to ubiquinone (Q), and from there to complexes III and IV, which pump protons (H) into the intermembrane space. Inhibitory interferences by etomoxir (Eto), ETFDH knockdown (siETFDH), TMEM126B knockout (sg126B), and hypoxia are depicted in blue.

Large-scale metabolomic studies and enzyme expression, as well as activity assays, are needed to further verify our hypothesis, and to generalize conclusions for cells others than monocytes. Oxygen consumption and mitochondrial fuel analysis under chronic hypoxia, and metabolite exploration, as well as mRNA expression regulation of glutamine and fatty acid-metabolizing enzymes, favor electron flow via ETF, rather than complex I or complex II, to maintain mitochondrial integrity (Figure 7).

## Figures and Tables

**Table 1:** List of primers.

Target	Forward (5′–3′)	Reverse (5′–3′)
ACC1	GCTTGCCTGACTTTTGATCCG	ACGTTATCCCCAAACCCAGG
ACLY	GATTTTGCGGGGTTCGTCG	TTGCCCGTCTGCTCTGAAAT
ETFA	ATTAGGTGACTGGCTGAGGC	GAAATCGTAGCAATGAGGCCG
ETFDH	GGAGTCCCTTATCTTTCCCTGG	ATCACCTGCCGGAAAGCAA
FASN	ATGAGCACCAACGACACGAT	CTATAGGCCGCAGCCTTCTC
GLS1	TATGGACATGGAACAGCGGG	CTGTCCTTGGGGAAAGGGTTT
GLS2	CTCCACCCACTAGAAAGCCAC	AAAGCTGGCTCCAGGGTTAG
GLUD1	ACAGTGGGCTGAAAACATCC	ATCACCAGGTTAAGCCATGC
GLUL	CTCTCGCGGCCTAGCTTTAC	CGGAGTTCACAGAGTAGGCG
GOT2	ATCCGTCCCATGTATTCCAA	TTCACTTCTTGCAGCCATTG
TBP	GGGCCGCCGGCTGTTTAACT	GGGCCGCCGGCTGTTTAACT

## Supplementary Materials

The following material is available online at https://www.mdpi.com/2073-4409/8/2/172/s1, Figure S1: Calculation of OCR ratios, Figure S2: Seahorse analyses and membrane potentials of control and siETFDH THP-1 cells, Figure S3: mRNA analyses of sg126B clones.
