# Supplementary material for: Chronic Hypoxia Enhances β-Oxidation-Dependent Electron Transport via Electron Transferring Flavoproteins
Source: Cells. 2019 Feb 18;8(2):172. doi: 10.3390/cells8020172 (PMC6406996; doi:10.3390/cells8020172)
Supplement: Supplementary file 1 [file cells-08-00172-s001.pdf]

Supplementary Figures:

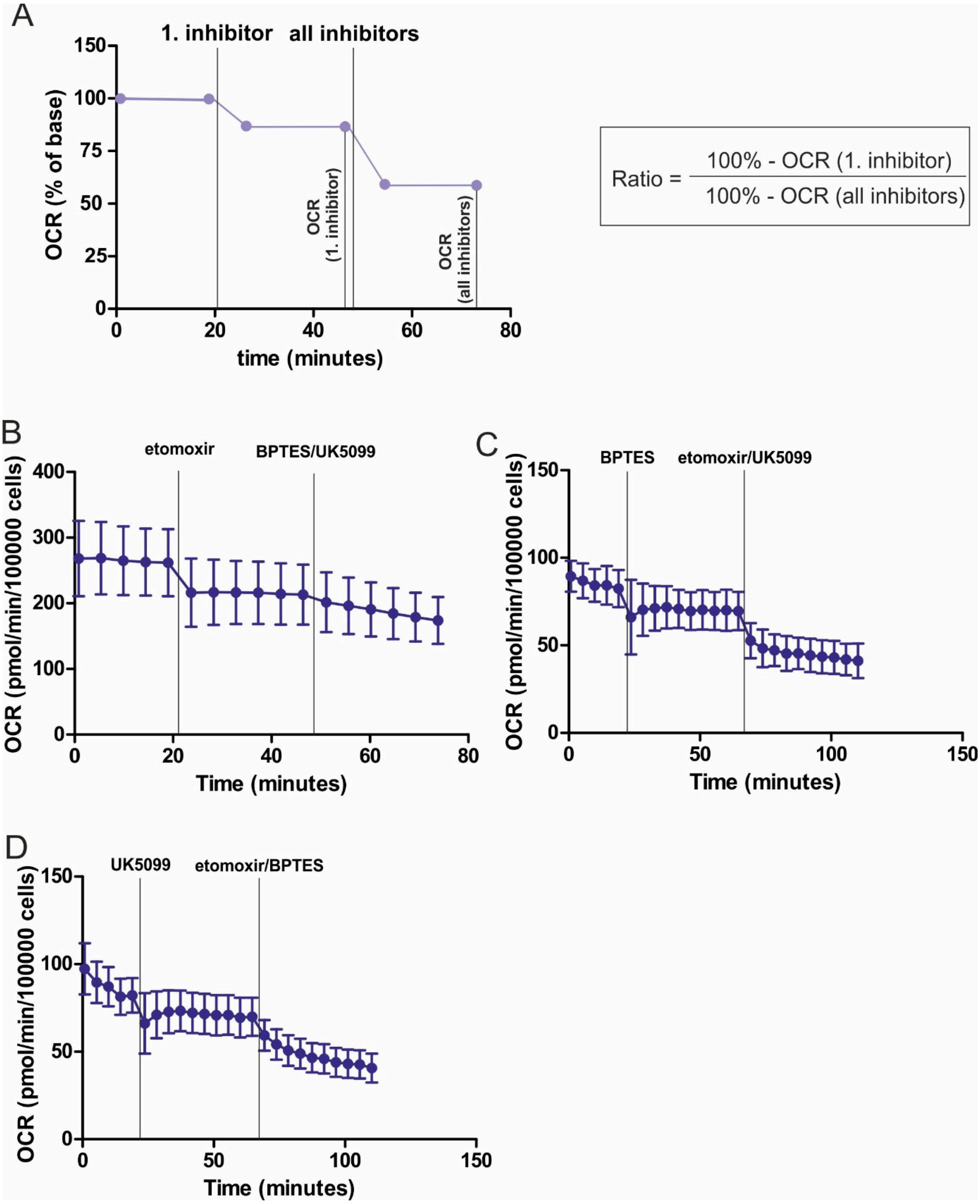

Figure S1: Calculation of OCR ratios

(A) To calculate the dependency of the electron transport chain on distinct substrates the baseline of oxygen consumption was set to 100%. After adding the first inhibitor the difference in OCR between baseline (without inhibitor) and the new baseline (with inhibitor) was determined. Thereafter, the two remaining inhibitors were added. Again, the difference in OCR between baseline (without inhibitor) and the new baseline (with all inhibitors) was determined. Then, the ratio of OCR with one inhibitor divided by the OCR with all inhibitors was calculated. (B-D) Seahorse measurements showing traces of OCRs with etomoxir, BPTES, and UK5099.

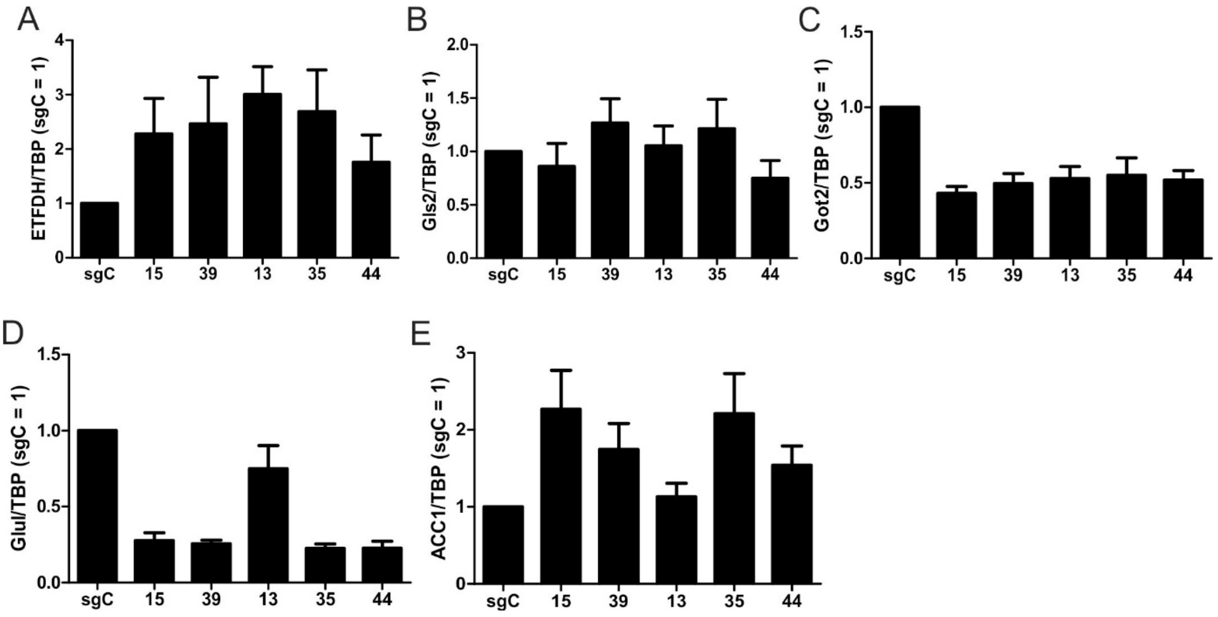

Figure S2: mRNA analyses of sg126B clones
(A-E) mRNA analyses of ETFDH (A), Gls2 (B), Got2 (C), Glul (D), and ACC1 (E) in sg126B and control cells (n = 5). TBP was used for normalization. Data are mean values ± SEM.
